# A common response to common danger? Comparison of animal and plant signaling pathways involved in cadmium sensing

**DOI:** 10.1007/s12079-012-0173-3

**Published:** 2012-08-04

**Authors:** Jagna Chmielowska-Bąk, Joanna Deckert

**Affiliations:** Department of Plant Ecophysiology, Institute of Experimental Biology, Faculty of Biology, Adam Mickiewicz University, ul.Umultowska 89, 61-614 Poznań, Poland

**Keywords:** Calcium ions, Cadmium, Nitric oxide, Mitogen-activated protein kinases, Reactive oxygen species, Transcription factors

## Abstract

Exposure to cadmium results in disturbances in cell homeostasis in all living organisms. The first response to stress factors, including cadmium, is activation of signal transduction pathways that mobilize cell defense mechanisms. The aim of this review is a comparison between the signaling network triggered by Cd in plants and animals. Despite differences in the structure and physiology of plant and animal cells, their cadmium signal transduction pathways share many common elements. These elements include signaling molecules such as ROS, Ca^2+^ and NO, the involvement of phospholipase C, mitogen-activated protein kinase cascades, and activation of transcription factors. Undoubtedly, both animals and plants also possess specific signaling pathways. In case of animals, Wnt/β-catenin, sonic hedgehog and oestorgen signaling are engaged in the transduction of cadmium signal. Plant specific signal transduction pathways include signaling mediated by plant hormones. The role of ethylene and jasmonic, salicylic and abscisic acid in plant response to cadmium is also discussed.

## Introduction

Cadmium is a non-essential heavy metal that is toxic to both plants and animals. Exposure to cadmium results in the generation of reactive oxygen species, membrane leakage, protein and DNA damage, perturbation in cell poliferation, and apoptosis (Deckert 2005; Lehotai et al. 2011; Sobkowiak and Deckert 2004; Vestena et al. 2011; Wang et al. 2011). In the case of mammals, cadmium has a carcinogenic effect (ATSDR 2011). The first response of organisms to the presence of cadmium in their environment is activation of the cell signaling network, which leads to changes in the metabolism indispensible for adaptation to unfavorable conditions. Despite the remarkable differences in the structure and physiology of plant and animal cells, their signal transduction pathways share many common elements. A question that arises is whether signal elements respond in a similar way to Cd exposure in organisms belonging to these two distant taxonomic groups. The aim of the present review is a comparison between the plant and animal signal transduction pathways activated by cadmium. The influence of Cd on signaling mediated by reactive oxygen species (ROS), calcium ions (Ca^2+^) and nitric oxide (NO), phospholipase C (PLC), mitogen-activated protein kinase (MAPK) cascades, as well as the expression of transcription factors will be reviewed. The last two sections will be dedicated to cadmium signal transduction pathways specific to animal and plant organisms.

### Cadmium uptake, accumulation and translocation

In the case of humans, cadmium is mainly absorbed through contaminated water, food or cigarette smoke, although other sources of intoxication are also possible (Mortensen et al. 2011; Yang et al. 2011). An interesting case was described recently in Saudi Arabia. A woman suffered from corneal injury after the usage of traditional eyeliner, which in a toxicology analysis exhibited elevated levels of lead and cadmium (Amry et al. 2011). After absorption, cadmium is transported into the liver, where it binds to small proteins such as glutathione or metallothioneins. Such complexes are released into the blood and might be excreted via urine or faeces, or accumulate in several target tissues, such as the kidneys, liver, pancreas, lungs, and bones (Crews et al. 2000; Thévenod 2009).

In plants, the roots are the main site of cadmium absorption and accumulation, although substantial levels of this heavy metal are also transported to the shoots. The root-to-shoot transfer of cadmium is mediated more by active (symplastic) than passive (apoplasitc) mechanisms (Lu et al. 2009; Vestena et al. 2011). Analysis using X-ray absorption spectroscopy showed that in the stems and leaves, Cd is bound to the oxygen and sulfur groups. Therefore, it is probable that during translocation between different plant organs, cadmium ions form complexes with small O- and S- containing molecules, such as organic acids and phytochelatins. It has been shown that treatment with citrate enhances a plant’s ability to transfer Cd from the roots to shoots (Chen et al. 2003). Moreover, over-expression of the plasma membrane H^+^-ATPase gene (*AHA1*) in *Arabidopsis thaliana* resulted in enhancement of the citrate concentration and secretion, together with an increase in the rate of cadmium root-to-shoot translocation (Hou et al. 2011; Rosa et al. 2004). The putative role of phytochelatins in cadmium transport has also been discussed, as these cysteine containing peptides were recently found in the xylem sap of oilseed rape (*Brassica napus*) subjected to low cadmium doses (Saathoff et al. 2011).

Cadmium, as a non-essential metal, is most likely taken up by cells through existing transport systems for essential bivalent cations. One of the candidates for cadmium transport are calcium channels. Treatment of Madin-Darby canine kidney (MDCK) cells with an activator of calcium channels, maitotoxin, resulted in enhanced Cd absorption (Olivi and Bessler 2000). Accordingly, pretreatment of Wistar rats with the calcium channel inhibitor verapamil caused a decrease in urinary Cd content, alleviation of cadmium-dependent oxidative stress, and kidney damage. However, verapamil had no effect on renal cortex cadmium content (Xu et al. 2010). Application of calcium channel inhibitors also modulated cadmium uptake in plants. Maize coleoptile segments treated with the calcium channel inhibitors verapamil and lantan chloride exhibited lower cadmium accumulation and diminished cadmium toxicity symptoms. Both calcium channel inhibitors also caused a significant decrease in cadmium concentration in *Suaeda salsa* roots (Kurtyka et al. 2011; Li et al. 2012). It has been suggested that the wheat transmembrane low-affinity-cation transporter (LCT1), which is known to transport Ca^2+^, is also engaged in Cd influx into plant cells. Expression of LCT1 in transgenic yeast resulted in a higher susceptibility to cadmium stress and higher Cd uptake (Clemens et al. 1998). However, later research showed that tobacco plants over-expressing the wheat *LCT1* gene accumulated less cadmium and exhibited a higher tolerance to this heavy metal than control lines. Therefore, it is unlikely that LCT1 is a main site of cadmium uptake in plant cells (Antosiewicz and Henning 2004).

Other candidates for cadmium influx site are ZIP transporters. These ZTR- and IRT-like proteins are responsible for iron, zinc, and manganese uptake. The expression of ZIP8 and ZIP14 in mouse fetal fibroblast cultures resulted in an increase in intracellular cadmium concentration and accelerated cell damage (Dalton et al. 2004; Girijashanker et al. 2008). Moreover, a connection between the induction of ZIP10 expression and Cd accumulation has been demonstrated in zebrafish (Chauchene et al. 2011). In the case of plants, the ZIP transporters IRT1, ZNT1, and ZNT2 are believed to play a role in cadmium uptake. The 35 S-IRT *Arabidospis* line, which is characterized by over-expression of the IRT1 gene, accumulated more cadmium in conditions of iron deficiency than in wild type plants (Connolly et al. 2002). The involvement of IRT proteins in cadmium transport is also supported by the fact that rice plants over-expressing IRT1 were characterized by higher cadmium content and exhibited higher sensitivity to this heavy metal than the wild type plants (Lee and An 2009). The ZNT1 and ZNT2 genes isolated from the hyperaccumulator *Thlaspi japonicum* conferred induced cadmium sensitivity in transgenic yeast (Mizuno et al. 2005).

An alternative candidate for cadmium transport are natural resistance-associated macrophage proteins (Nramp). Injection of human Nramp2, described also as DCT1 or DMT1, to *Xenopus* oocytes resulted in enhanced cadmium transport. Noteworthy, absorption of the heavy metal was strongly dependent on pH (Okubo et al. 2003). Nramp proteins might be also engaged in cadmium uptake in plants. Characterization of rice Nramp-1 transporter revealed that this protein is located in plasma membrane and that its over-expression results in enhanced accumulation of Cd in rice leaves (Takahashi et al. 2011). Nramp transporters are probably also involved in cadmium vacuolar compartmentalization. There are several facts that support this concept. Firstly, it has been observed in *Arabidpsis halleri* and *Thlaspi caerulescens* that Nramp3 and Nramp4 proteins are localized in the vacuole membrane. Secondly, the two mentioned cadmium hyperaccumulators, *Arabidpsis halleri* and *Thlaspi caerulescens*, exhibit higher expression levels of Nramp transporters when compared to more sensitive plant species. Lastly, the double mutant *Arbidopsis thaliana nramp3nramp4* displays a hypersensitivity to zinc and cadmium without a change in the cellular levels of these heavy metals (Chiang et al. 2006; Oomen et al. 2009). Another transporters which might be involved in vacuolar sequestration of cadmium are P-ATPases. Expression of one P_1B_-ATPase, AtHMA3, which is found in the vacuolar membrane, increased cadmium tolerance in yeast Cd sensitive *Δycf1* mutant (Gravot et al. 2004).

The above-mentioned studies show that cadmium ions are most likely “hitch-hikers” which get into the cell via channels destined for other ions. However, it has been shown that the Ganges ecotype of *Thlaspi caerulescens* possesses a high affinity transporter for cadmium ions. The author raises the question of the evolutionary meaning of possessing transporters specific for non-essential heavy metals (Lombi et al. 2001).

### The role of reactive oxygen species in cadmium signal transduction

An increase in the level of reactive oxygen species is one of the most universal responses to cadmium stress (Hsu and Kao 2007a; Kippler et al. 2012; Kopyra and Gwóźdź 2003; Lehotai et al. 2011; Pytharopoulou et al. 2011; Vestena et al. 2011; Wang et al. 2011). There are several mechanisms that lead to Cd-induced accumulation of ROS. It is postulated that cadmium replaces enzyme-bound-metals that display oxidation-reduction activity such as Fe or Cu. The released metals lead to ROS generation through a Fenton reaction (Casalino et al. 1997). It has been also shown that Cd-induced ROS generation is dependent on the activation of NADPH oxidase in both plant and animal cells. This membrane-bound enzyme produces O_2_^•^, which is then rapidly converted into H_2_0_2_ (Chen et al. 2011b; Cho et al. 2012; Chou et al. 2012; Garnier et al. 2006; Rodríguez-Serrano et al. 2006). Another source of ROS are mitochondria alerted by Cd (Garnier et al. 2006). Cadmium might also lead to a decrease in antioxidant enzyme activity and in this way contribute to an increase in ROS cellular levels (Cho and Seo 2005; Gzyl et al. 2009; Ognjanović et al. 2010; Romero-Puertas et al. 2007). Reactive oxygen species interact with various cellular compounds and lead to their damage. The negative impact of ROS on cellular homeostasis includes changes in membrane permeability, the inactivation of enzymes, and an increased rate in DNA mutation. It has been demonstrated in two cultivars of *Arabidopsis thaliana* differing in their sensitivity to cadmium that oxidative stress is major contributor to the Cd-dependent phytotoxicity (Cho and Seo 2005). Despite their harmful action, ROS might also play a positive role in response to stress conditions. Some reactive oxygen species act as signaling molecules and in this mode mediate activation of defense mechanisms (Ghouleh et al. 2011; Bhattacharjee 2005). Experiments with the use of microarrays revealed that hydrogen peroxide regulates the expression of 193 genes in human lymphoblastoid TK9 cells, 1404 genes in human colon cancer cells, and 680 genes in *Arabidposis* seedlings (Briedé et al. 2010; Platel et al. 2010; Wang et al. 2006). It has been recently suggested that the signal can be transduced not only by ROS themselves but also by oxidized fragments of proteins damaged by oxidative stress. The derived peptides could act in more a specific way, as they contain information about the organelle subjected to stressful conditions and the type of ROS produced (Mǿller and Sweetlove 2010).

In terms of response to cadmium stress, the role of reactive oxygen species in cellular signaling in the initiation of apoptotic processes has been most commonly examined. The involvement of ROS in apoptosis has been observed in splenocytes, rat testes, astrocytes and proximal tubular cells, human hepatoma, neuroblastoma and retinal pigment epithelial cells, tomato, tobacco, and *Arabidopsis* suspension culture (Catterjee et al. 2009; Garnier et al. 2006; Oh and Lim 2006; Kalariya et al. 2009; Kim and Soh 2009; De Michele et al. 2009; Wang et al. 2011; Yakimova et al. 2006; Yang et al. 2008). In animal cells, two apoptosis signaling pathways can be distinguished: the first one engages the activation of caspases and the second one is caspase-independent (Lee and Thévenod 2008). There is evidence that reactive oxygen species are implied in both signaling pathways. The blockage of Cd-induced ROS accumulation in human hepatoma cells by pretreatment with N-acetylcysteine (NAC) resulted in the inhibition of apoptosis through reversion of capsase-8, -3 and -9 activation, accompanied by the inhibition of Bid and Bax protein cleavage and hampered Cyt c release (Oh and Lim 2006). On the other hand, in rat testes subjected to cadmium stress, ROS has been shown to be engaged in poly ADP-ribose polymerase-1 (PARP-1) induction, translocation of the apoptosis-inducing factor (AIF) from mitochondria to nucleus, and, in consequence, initiation of the caspase-independent apoptotic pathway (Kim and Soh 2009). Activation of caspase independent apoptotic processes in response to cadmium exposure has been also demonstrated in rat astrocytes. This process has been mediated by an increase in intracellular calcium levels, which in turn lead to ROS generation (Yang et al. 2008). It seems that proteins which resemble animal caspases also play a role in Cd- induced apoptosis in plants. Treatment of a tomato suspension culture subjected to cadmium stress with human caspase inhibitors caused a significant reduction in the cell death rate. The same experiments showed that the apoptotic signaling activated by CdSO_4_ in a tomato suspension engages reactive oxygen species, calcium ions, calmodulin, phospholipid signaling, protein kinases, and one of the plant hormones—ethylene (Yakimova et al. 2006). Similar results were obtained in a tobacco suspension culture. Treatment with CdCl_2_ caused an increase in ROS production, which led to an increased rate of cell death. The generation of reactive oxygen species was dependent on phospholipase C and calmodulin activity (Garnier et al. 2006).

Another cadmium-induced process that is dependent on ROS accumulation is autophagy. This type of programmed cell death consists in the self-digestion of cells. It is still under discussion if autophagy is a cell defense strategy, mode of suicide, or both (Edinger and Thomson 2004). There is evidence that cadmium causes autophagy in both animal and plant cells, and that the process in both cases is mediated by reactive oxygen species (Son et al. 2011; Yang et al. 2009; Zhang and Chen 2010). In mesangial cells, cadmium induces the generation of reactive oxygen species, which in turn activate glycogen synthase kinase-3β (GSK-3β) responsible for the initiation of autophagy (Yang et al. 2009). The sequence of events which lead to mouse epidermal skin cells autophagy consists of ROS generation, phosphorylation of LKB1 and AMPKα proteins, and the formation of LC3-II (Son et al. 2011).

Reactive oxygen species are involved in Cd-induced tumorigenesis. It has been reported that hydrogen peroxide and superoxide anion are required for increased expression of c-*fos*, c-*jun* and c-*myc* proto-oncogenes in BALB/c-3 T3 cells (Joseph et al. 2001). Reactive oxygen species are also engaged in tumor angiogenesis—a critical process for tumor initiation and growth (Jing et al. 2012).

There is crosstalk between ROS signaling and other signal transduction pathways. It has been shown that ROS generation is dependent on Ca^2+^ signaling in both animal and plant cells (Yang et al. 2008; Rodríguez-Serrano et al. 2006). In rice leaves, Cd-dependent H_2_O_2_ accumulation is mediated also by phosphatidylinositol 3-phosphate and nitric oxide (Hsu and Kao 2007b). Reactive oxygen species, on the other hand, work upstream from other signaling elements such as kinases. Experiments conducted with the use of an ROS scavenger have shown that reactive oxygen species activate or/and induce expression of extracellular signal-regulated kinase (ERK), LKB1-AMP-activated kinase (AMPK) and *c-Jun* N-terminal kinase (JNK) in mammalian cells as well as MPK3 and MPK6 in *Arabidopsis* cells (Jing et al. 2012; Kalariya et al. 2009; Kim et al. 2005; Liu et al. 2010; Son et al. 2011).

### Calcium signaling in response to cadmium

Calcium ions are one of the most important and conserved signal transduction elements found in all living organisms. The mode of action consists in the regulation of protein activity via modifications of its charge and conformation. There is a vast number of calcium sensor proteins, such as highly conserved calmodulin (CAM), plant calmodulin-like proteins (CLM), calcineurin B-like proteins (Hashimoto and Kudla 2011), and Ca^2+^ dependent protein kinases or neuronal Ca^2+^ sensors (NCS) specific to animal cells (Clapham 2007). After binding to Ca^2+^, these calcium sensors are able to interact with other cellular components and modify their structure or/and activity. The targets for calmodulin include kinases, ions transporters, G-proteins, cytoskeleton compounds, and transcription factors. The calcium signal is strictly dependent on the time and spatial course of Ca^2+^ accumulation. To control calcium action, cells have developed an extended system of Ca^2+^ compartmentalization, chelation, and excretion (Clapham 2007; Hashimoto and Kudla 2011; Snedden and Fromm 1998).

There are several difficulties in conducting research concerning the involvement of calcium ions in response to cadmium stress. The molecules possess the same charge, and the ion radius Cd^2+^ and Ca^2+^ are very similar. Therefore, both Cd^2+^ and Cd^2+^ tend to bind to similar proteins, which makes it difficult to find a fluorescent probe specific only for calcium ions. Experiments with the use of calcium channel inhibitors are also complicated because, as has been described in previous sections, cadmium is taken up by cells through the calcium transport systems.

In spite of the previously-mentioned difficulties, there is convincing evidence that cadmium causes an increase in cytosolic calcium levels in various animal cell types (Faurskov and Bjerregaard 2002; Misra et al. 2002; Shankar et al. 1992; Smith et al. 1989; Yamagami et al. 1998; Yang et al. 2008; Ye et al. 2007). The observed Cd-induced intracellular calcium release depends on phospholipase C (PLC) activation and an increase in cellular inositol triphosphate concentration. Interestingly, cadmium ions had no effect on the Ca^2+^ intracellular levels in human epidermoid carcinoma, rat embryo fibroblasts, and rat aortic smooth muscle cells. These results suggest that the phenomena of calcium mobilization by cadmium ions is dependent on the organism and type of cell (Smith et al. 1989). An increase in intracellular calcium levels is essential for various Cd-induced processes, such as cell transformation, tumorigenesis, and apoptosis. It has been shown in mouse tumor-derived cell lines that accumulation of Ca^2+^, together with ROS generation, is necessary for activation of *c-fos*, *c-jun* and *c-myc* proto-oncognes (Joseph et al. 2001). Intracellular calcium influx is also engaged in Cd-induced apoptosis processes (Chen et al. 2011a; Wang et al. 2008; Ye et al. 2007). In coronary neurons, the Cd-dependent apoptosis signaling network includes an increase in Ca^2+^ influx, which leads to the stimulation of CaMPKII. CaMPKII in turn activates subsequent signaling elements—mitogen-activated kinases (MAPKs) and mTOR pathway (Chen et al. 2011a).

The changes in cytosolic calcium levels in response to cadmium treatment in plants seem to depend on the plant species and organ. An increase in cytosolic Ca^2+^ concentration has been reported in tobacco cells and rice roots, while a significant decrease has been shown in *Arabidopsis thaliana* root hairs (Fan et al. 2011; Garnier et al. 2006; Yeh et al. 2007). As has been described in previous sections, calcium signaling plays a role in Cd-induced apoptosis in tobacco and in an *Arabidposis* suspension culture (Garnier et al. 2006; Yakimova et al. 2006). The involvement of calcium ions in plant response to cadmium can be also presumed on the basis of the fact that CdCl_2_ caused an increase in the expression of genes coding calmodulin-like protein in black night shade and of the calcium-binding protein HvC2d1 in barley plants (Oulhajd et al. 2006; Xu et al. 2009).

### Nitric oxide signaling and cadmium stress

Since the discovery that nitric oxide plays an important role in the regulation of the cardiovascular system in the late 1980s, this molecule has had an astonishing career in the biological sciences. Its popularity is reflected by the fact that in 1992 NO was chosen as the Molecule of the Year by the leading scientific journal *Science* (Hasanuzzaman et al. 2010). Nitric oxide is a signaling molecule that can act directly through regulation of protein activity by S-nitrosylation and nitrotyrosylation, or indirectly by modification of other signaling pathways, such as protein kinases or calcium signaling (Krasylenko et al. 2010). Among the processes which are controlled by nitric oxide signaling in animals, cell proliferation, embryogenesis, cardiovascular tension maintenance, neurotransmission, immune defense, apoptosis, and the regeneration of lower metazoans can be listed (Colasanti et al. 2010; Moncada and Higgs 2006). In plants, NO also regulates a vast number of processes, such as seed germination, plant growth, tissue differentiation, flowering, seed maturation, senesces, the initiation of programmed cell death, as well as responses to biotic and abiotic stress factors (Hasanuzzaman et al. 2010; Krasylenko et al. 2010). There are some discrepancies concerning the pattern of NO accumulation in response to Cd. It has been suggested that short-time cadmium treatment causes the induction of NO production, while longer treatment periods lead to a decrease in the level of nitric oxide. Such NO production kinetics have been observed in mouse peritoneal macrophages characterized by an increase in NO concentration 6, 18 and 24 h after cadmium treatment and depletion in prolonged treatment (Ramirez et al. 1999). A similar effect has been observed in two independent experiments performed on pea roots. During short-term exposure to Cd ions (24 and 48 h of treatment), an enhancement in NO production was observed, while in plants treated for 14 days, the levels of nitric oxide where lower than in the control (Lehotai et al. 2011; Rodríguez-Serrano et al. 2006). However, there is evidence that NO production can be inhibited even shortly after exposure to cadmium, and, conversely, elevated nitric oxide amounts were observed in organisms treated with Cd for relatively long time periods (Groppa et al. 2008; Mahmood et al. 2009; Xiong et al. 2009). Thus, it seems that the pattern of nitric oxide accumulation in reaction to cadmium depends on the organism, cell type, metal concentration, and treatment duration (for reference see Table 1). There are also conflicting data concerning the involvement of nitric oxide in plant defense against cadmium stress. On the one hand, there is evidence that NO can diminish Cd-induced oxidative stress by stimulating the antioxidant system. This molecule has been also shown to promote hemicelluloses and pectin synthesis, which may facilitate cadmium immobilization (Arasimowicz-Jelonek et al. 2011). On the other hand, nitric oxide amplifies cadmium uptake in *Arabidopsis* and tobacco BY-2 cells (Besson-Bard et al. 2009; Ma et al. 2010). Moreover, nitric oxide causes the nitrosilation of phytochelatins and participates in the initiation of apoptosis (Arasimowicz-Jelonek et al. 2011). It is still under discussion whether these effects promote cadmium tolerance or, conversely, lead to the augmentation of Cd toxicity.

**Table 1:** Influence of cadmium on NO generation

Organism	Effect	Cadmium concentration	Treatment duration	References
Animals				
Haemocytes of mussel	↑	5, 10 and 50 μM	1 h	Dailianis 2009
*Mytilus galloprovincialis*				
Mouse peritoneal macrophages	↑	10 μM	6, 18 and 24 h	Ramirez et al. 1999
Human endothelial cells	↓	1 and 5 μM	8 h	Kolluru et al. 2006
Human macrophages culture	↑	0,4–0,7 μM	48 h	Hassoun and Stohs 1996
Mouse peritoneal macrophages	↓	10 μM	72 h	Ramirez et al. 1999
Mouse peritoneal macrophages	↑	15 ppm	2 months	Ramirez and Gimenez 2003
Plants				
Tobacco BY-2 cells	↑	150 μM	2,4,6,8 and 12 h	Ma et al. 2010
*Arabidopsis thaliana* roots	↑	200 μM	7 h	Besson-Bard et al. 2009
Barley roots (*Hordeum vulgare*)	↑	1 mM	24 h	Valentovičová et al. 2010
Rice roots (*Oryza sativa*)	↓	100 μM	24 h	Xiong et al. 2009
Pea roots (*Pisum sativum*)	↑	100 μM	24 and 48 h	Lehotai et al. 2011
Arabidopsis suspension cultures	↑	150 μM	48 h	De Michele et al. 2009
Wheat roots (*Triticum aestivum*)	↑	10 μM	72 h	Mahmood et al. 2009
Soybean cell suspension (*Glycine max*)	↑	4 μM and 7 μM	72 h	Kopyra et al. 2006
Wheat roots (*Triticum aestivum*)	↑	100 μM	5 days	Groppa et al. 2008
Pea leaves *(Pisum sativum*)	↓	50 μM	14 days	Rodriguez-Serrano et al. 2009
Pea roots (*Pisum sativum*)	↓	50 μM	14 days	Rodriguez-Serrano et al. 2006
Pea leaves (*Pisum sativum*)	↓	50 μM	14 days	Barroso et al. 2006
Wheat roots (*Triticum aestivum*)	↑	1 μM	28 days	Mahmood et al. 2009

Animal cells are also affected by changes in NO concentration. A lowering of NO concentration in response to cadmium might lead to disturbances in various processes, such as angiogenesis and immune defense (Kolluru et al. 2006; Thévenod 2009). Nitric oxide might also play a protective role during cadmium stress. The NO-releasing prodrug V-PYRRO/NO alleviates Cd cytotoxicity in rat liver cells, most probably through the induction of metallothioneine (MT) synthesis. The chemical agent was also shown to inhibit *c-Jun* N-terminal kinase (JNK1/2). As the activation of JNK1/2 is connected with induction of apoptosis, NO released from V-PYRRO/NO might arrest the Cd-induced cell death in rat liver (Qu et al. 2005).

### The role of phospholipase C in response to cadmium action

Phospholipase C is a membrane-bound enzyme that catalyses the hydrolysis of phosphoinositol 4,5-biphosphate (PIP_2_). In the reaction, two second messengers, diacyloglycerol (DAG) and inositol 1,4,5 triphosphate (IP_3_), are formed. An accumulation of IP_3_ leads to the release of Ca^2+^ from intracellular stores, while DAG functions as an activator for protein kinase C (PKC). There are several isoformes of PLC, among which the δ isoenyzme is present in fungi, plants and animals (Ochocka and Pawelczyk 2003).

As has been described in the section concerning calcium signaling, the results of several experiments imply that in animal cells, cadmium stimulates phospholipase C, which leads to an increase in IP_3_ levels and the release of Ca^2+^ (Faurskov and Bjerregaard 2002; Misra et al. 2002; Smith et al. 1989; Yamagami et al. 1998). The activation of phospholipase C might also lead to the accumulation of DAG and the induction of protein kinase C activity. In fact, an increase in PKC activity in response to cadmium treatment has been reported in the freshwater crab *Sinoptoman yangtsekiense* (Li et al. 2011). Studies with the use of protein kinase C inhibitors showed that PKC plays a significant role in various processes induced by cadmium action. These processes include accumulation of the transcription factor Nrf2 in the nucleus of astorcytoma cells, perturbations in calcium homeostasis in osteasarcoma cells, actin gluthionylation, and protein carbonylation in the haemocytes of the mussel *Mytilus galloprovincialis* (Dailianis et al. 2009; Lawal and Ellis 2011; Long 1997).

Phospholipase C might perform protective functions under cadmium stress, as a transgenic fibroblast that overexpressed the *PLCβ-1* gene exhibited greater viability under cadmium treatment when compared to control cells (Lee et al. 2000). Moreover, PLC is engaged in the stimulation of plants under low cadmium doses. Barley seedlings treated with Cd in a concentration of 5 × 10^−8^ M exhibited higher levels of chlorophyll and cytokinins. The application of various inhibitors showed that this effect was mediated by DAG, PKC, and MAPK (Kovács et al. 2009). In a tomato suspension culture, on the other hand, PLC has been shown to mediate Cd-induced cell death. Application of the PLC inhibitor neomycin significantly decreased the number of dead cells (Yakimova et al. 2006).

### MAPK cascades and Cd^2+^

Mitogen-activated protein kinases (MAPKs) form a conserved signal transduction mechanism that can be found in all eukaryotic organisms. The MAPK cascade consists of at least three elements: MAPKKK, MAPKK, and MAPK. The activation of MAPKKK leads to the phosphorylation of MAPKK, which in turn phosphorylates MAPK. MAPKs have the ability to phosphotylate, and in this way regulate the activity of various cellular components, such as other protein kinases, proteins associated with cytoskeleton, and transcription factors. In the *Arabidopsis* genome, 60 MAPKKKs, 10 MAPKKs, and 20 MAPKs have been found. Taking into account the fact that MAPKKKs can interact with various MAPKKs, and different MAPKKs can activate various MAPKs, the mitogen-activated protein kinase cascades form an extensive and complicated signaling network. MAPK cascades are engaged in the regulation of various processes, including the response to stress factors (Chang and Karin 2001; Nakagami et al. 2005).

In mammalian cells, MAPKKs (also called MEKs or MKKs) work upstream from MAPKs, which include extracellular signal-related kinase (ERK), Jun amino-terminal kinase (JNK), and p38 proteins (Chang and Karin 2001). The majority of reports state that Cd activates all of the above-mentioned MAPKs (Chen et al. 2008; Jing et al. 2012; Jung et al. 2008; Kalariya et al. 2009; Kim et al. 2005; Park et al. 2009; Valbonesi et al. 2008). However, in experiments performed on human embryonic kidney cells, the activity of JNK was stimulated only at higher cadmium concentrations (50 μM), while treatment with low Cd doses (0.5 μM) caused a decrease in its activity (Hao et al. 2009). Moreover, in microcultures of limb bud cells isolated from mouse embryos, cadmium caused phosphorylation of JNK, while the level of phosphorylated ERK and p38 remained unchanged (Liu and Kapron 2010). There is extensive evidence that activation of MAPKs in response to cadmium is mediated by reactive oxygen species. The application of ROS scavengers to human retinal pigment epithelial cells treated with Cd reversed the Cd-induced phosphorylation of JNK, ERK1/2 and p38 (Kalariya et al. 2009). A connection between ROS generation and the activation of JNK and/or ERK has been also observed in mouse macropaghes and in human neuronal and lung epithelial cells exposed to Cd (Chen et al. 2008; Jing et al. 2012; Kim et al. 2005). Moreover, both H_2_O_2_ and Cd caused a similar pattern of JNK, ERK, and p38 activation in extravillous trophoblast cells (Valbonesi et al. 2008).

The nomenclature of plant mitogen-activated protein kinase network might be confusing, although some attempts to clarify the system has been made. Generally, it has been proposed to adopt name MPKs for MAPKs and MKKs for MAPKKs. Plant MPKs include tobacco SIPK, WIPK and NTF, alfalfa SAMK and SIMK and rice BWMK kinases. Some examples of plant MKKs are tobacco MEK and SIPK and alfalfa PRKK and SIMKK (Ichimura and MAPK Group 2002). It has been shown that various components of plant MAPK cascades are stimulated by cadmium. Exposure to Cd resulted in the activation of MPK3 and MPK6 in *Arabidposis* as well as of SIMK, MMK2, MMK3, and SAMK in alfalfa (Jonak et al. 2004; Liu et al. 2010). Moreover, rice plants subjected to cadmium stress exhibited elevated expression of the *OsBWMK1*, *OsMSRMK2*, and *OsEDR1* genes (Agrawal et al. 2002, 2003; Kim et al. 2003). The response of MAPK cascades to cadmium action is very rapid, as the induction of gene coding MAPKKK has been observed as early as 15 min after the application of Cd, while the enhanced phosphorylation of MAPKs has been noted even after 10 min of cadmium treatment (Jonak et al. 2004; Kim et al. 2003). It is probable that, just like in animals, the activation of MAPKs in plants is mediated by reactive oxygen species. Pre-treatment of *Arabidopsis* plants with the ROS scavenger, gluthatione, resulted in the attenuation of Cd-induced MPK3 and MPK6 phosphorylation (Liu et al. 2010). Similar results were obtained in experiments performed on rice. Cadmium increased the activity of 40 kDa and 42 kDa MAPKs. This effect was reversed by pre-treatment with a ROS scavenger, as well as with an NADPH oxidase inhibitor, suggesting that the activation of MAPK was dependent on ROS generated by this membrane-bound enzyme. Stimulation of MAPKS was also connected with calcium dependent protein-kinases (CDPK), phosphoinositol 3-kinase (PI3), and the functional state of mitochondria (Yeh et al. 2007).

### Cadmium-induced transcription factors

The activation of signaling pathways leads to changes in the activity of transcription factors (TFs), which in turn regulate the expression of various genes. It has been calculated that animals possess approximately 860 different transcription factors per species which form 4.65 % of the genomic content. In the case of plants, these numbers come to 590 and 2.12 % respectively. Transcription factors are classified into several families, frequently named after their DNA binding domains (Charoensawan et al. 2010).

In animal cells, several genes induced by heavy metals possess specific sequences in their promoter, called metal responsive elements (MRE). These sequences are recognized by the MTF-1 transcription factor (metal-regulatory or MRE-binding transcription factor). The DNA-binding ability of MTF-1 is dependent on the concentration of zinc ions. It has been suggested that other heavy metals, including cadmium, regulate MTF-1 activity through the release of zinc ions from intracellular stores (Lichtlen and Schaffner 2001). Another hypothesis states that several factors, such as zinc, cadmium, hydrogen peroxide, or heat shock, mediate the transfer of MTF-1 from cytoplasm to nucleus (Lichtlen and Schaffner 2001). Indeed, treatment of mouse hepatoma cells with cadmium increased the amount of MTF-1 in the nucleus and decreased its concentration in cytoplasm (Smirnova et al. 2000). A similar phenomenon has been observed in the case of another transcription factor, Nrf2. Treatment of rat liver and mouse hepatoma cells with CdCl_2_ resulted in an increase in Nrf2 levels. The induced TF was found mainly in the nucleus (Casalino et al. 2007; Stewart et al. 2003). Accumulation of Nrf2 under Cd treatment is most probably dependent on the ability of cadmium to stabilize this protein and prolong its half-life (Stewart et al. 2003). Nrf2 plays a role in Cd-dependent stimulation of oxygenase-1 and cytochrome p4502A5 (Abu-Bakar et al. 2004; Alam et al. 2000).

A sequence similar to animal MRE, called PvSr2, has been identified in beans. Transgenic tobacco plants into which the reporter *GUS* transgene was introduced under the control of the PvSR2 promoter, exhibited an elevated expression of the *GUS* gene in response to Cu^2+^, Zn^2+^, Hg^2+^, and Cd^2+^ treatments (Qi et al. 2007). However, to our knowledge no transcription factor associated with the above-mentioned MRE sequence has been identified so far. It is, however, known that in plants, TFs belonging to the AP2/EREBP, MYB, WRKY, and bZIP families are involved in the response to cadmium action (DalCorso et al. 2010). BrCdR15 isolated from *Brassica juncea* is an example of a transcriptional factor belonging to the bZIP family that is induced by cadmium. Tobacco and *Arabidposis* plants that overexpressed BjCdR15 exhibited a higher tolerance to Cd and a higher Cd content in their shoots. The fact that this TF regulates the synthesis of phytochelatines and metal transporters suggests that BjCdR15 confers cadmium tolerance through regulation of cadmium uptake and root-to-shoot translocation (Farinati et al. 2010). Comprehensive analysis of the expression profiles of 163 transcription factors belonging to the MYB family in *Arabidposis thaliana* showed that approximately 20 % of them respond to cadmium treatment (Yanhui et al. 2006). Another source of information about transcription factors engaged in transduction of the cadmium signal are global analyses of gene expression. Analysis of the expression profiles of genes regulated by Cd showed that this metal induces genes which code OsDREB1A, OsDREB1B, and WKRY09 in rice. Another experiment showed that in *Arabidopsis thaliana* genes which code transcription factors containing ATAF, DREB2A, bZIP and WRKY motifs were induced by treatment with cadmium ions. At the same time, several transcription factors belonging to the AP2/ERF superfamily were down-regulated by Cd in *Solanum turvom* (Ogawa et al. 2009; Suzuki et al. 2001; Yamaguchi et al. 2009)

### Animal specific signaling

One of the characteristic effects of cadmium action, that is specific to animal organisms, is induction of cell proliferation which leads formation of cancer tumors (Deckert 2005). It has been suggested that the carcinogenic effect of Cd is connected with its ability to mimick Wnt/β-catenin signaling. In mammalian cells β-catenin plays dual role—it performs structural functions by bridging E-cadherin to α-catenin in adherens junctions and acts as signaling molecule that can regulate expression of various genes including proto-oncogenes. The Wnt/β-catenin signaling includes several steps: binding of Wnt to receptor, inactivation of β-catenin “destruction” complex, increase in cytoplasmic and nuclear β-catenin levels and finally interaction of β-catenin with T-cell specific factors/lymphoid enhancer binding factor (TCF/LEF), which leads to the activation of target genes (Berthon et al. 2012; Thévenod 2009; Weisberg et al. 2003). Several studies show that cadmium causes loss of adherens junctions integrity which leads to the translocation of β-catenin from membrane to cytoplasm and nuclei (Chakraborty et al. 2010; Prozialeck et al. 2003; Thompson et al. 2008). Translocation of β-catenin to nucleus results in formation of β-catenin/TCF4 complexes. These complexes are engaged in activation of Wnt signaling target genes, *c-myc*, *cyclin D1* and *Abcd1a* (Chakraborty et al. 2010). In contrast to the described studies, experiments performed on mice showed that prenatal cadmium treatment caused down-regulation of Wnt/β-catenin signaling pathways in thymus of the offspring. This effect was exhibited by the decrease in *Wnt10b* gene expression, increase in cytoplasmic phospho-β-catenin, predestined for degradation, and decrease in active β-catenin in nucleus (Hanson et al. 2010).

Sonic hedgehog (shh) signaling is an example of another specific animal signaling pathways alerted by cadmium. The Shh receptors consist of two transmembrane proteins: patched (ptc) and smoothened (smo). Binding of Shh protein to the receptor results in abolishment of ptc inhibitory effect on smo. Activated smo transduces the Shh signal to other signaling elements such as Gil proteins (Benson et al. 2004). Two independent experiments showed that prenatal cadmium treatment causes decrease in Shh signaling in mice fetuses and thymus of mice offspring. Interestingly both studies imply that, despite the observed decrease in Shh signaling, Shh and Gil protein levels were unchanged. It is suggested that the decrease in Shh signaling pathway is responsible for developmental anomalies such as postaxial ectrodactyly and abnormal thymocyte development (Hanson et al. 2010; Scott et al. 2005).

The influence of cadmium on oestrogen signaling has been recently profoundly reviewed in Silva et al. 2012. Generally, it has been demonstrated in several studies performed on human breast cancer cell lines, that cadmium is able to bind to membrane and nuclear oestrogen receptors (ER) and activate oestrogen response. This response includes activation of AKT and ERK1/2, induction of *c-myc*, *c-jun* and *c-fos* proto-oncogenes and stimulation of cell proliferation (Silva et al. 2012).

### Specific plant response

Plants also possess regulatory mechanisms that are specific to this taxonomic group. These mechanisms include signaling mediated by plant hormones (McSteen and Zhao 2008). Ethylene is frequently called a plant stress hormone, as an increase in its levels has been observed under various unfavorable conditions, including cadmium stress (Arteca and Arteca 2007; Rodríguez-Serrano et al. 2006). Masood et al. 2012 showed that an increase in ethylene concentration in mustard plants subjected to Cd is correlated with an increase in 1-aminocyclopropane-1-carboxylic acid synthase activity (ACS). ACS is a key enzyme in the ethylene synthesis pathway (Masood et al. 2012). Several functions in plant response to cadmium stress are ascribed to ethylene action. This hormone mediates Cd-induced accumulation of H_2_O_2_. Bean and onion plants treated with CdCl_2_ and STS, an inhibitor of ethylene perception, showed a significant decrease in H_2_O_2_ levels when compared with plants treated only with CdCl_2_ (Maksymiec 2011). A tomato mutant with the antisense *ACS* gene also exhibited a reduction in ethylene synthesis accompanied by reduced hydrogen peroxide levels under cadmium stress (Liu et al. 2008). Ethylene is engaged in the induction of apoptosis. Application of the ethylene synthesis inhibitor AVG to tomato suspension cultures subjected to CdSO_4_ resulted in a decreased number of dead cells (Yakimova et al. 2006). This plant hormone also has a protective effect against cadmium toxicity on photosynthesis. Treatment of mustard plants with the ethylene donor, ethephon, alleviated the inhibitory effect of cadmium on the activity of Rubisco—a key enzyme in the photosynthesis process (Masood et al. 2012).

Among plant hormones, in addition to ethylene, salicylic (SA), jasmonic (JA), and absicis acid (ABA) might play a role in cadmium signal transduction. Accumulation of salicylic acid under cadmium stress has been noted in pea, maize and *Arabidopsis* plants (Krantev et al. 2008; Rodríguez-Serrano et al. 2006; Zawoznik et al. 2007). The majority of reports state that SA protects plants against the toxic effects of cadmium. Exogenous application of SA caused alleviation of Cd-induced oxidative stress, which was most probably connected with stimulation of the antioxidant system (Krantev et al. 2008; Panda and Patra 2007; Zhang et al. 2011). It is possible that salicylic acid also activates other ROS scavenging mechanisms, as in mustard a SA-dependent decrease in Cd-induced H_2_O_2_ levels was accompanied by inhibition of the activity of antioxidant enzymes (Ahmed et al. 2011). Interestingly, experiments performed on rice imply that the protective role of SA is dependent on SA-induced generation of hydrogen peroxide. Therefore, SA might play a biphasic role in ROS metabolism. On one hand it mediates H_2_O_2_ accumulation through stimulation of NADPH oxidase activity, on the other hand the SA-dependent H_2_O_2_ production is essential for activation of antioxidant enzymes (Chao et al. 2010). The role of SA in ROS generation is also supported by the fact that SA-deficient *Arabidopsis* mutants exhibited decreased levels of H_2_O_2_ and diminished lipid peroxidation in response to CdCl_2_ when compared with the wild type plants. Moreover, transgenic plants showed, in contrast to the wild type, induction of guaiacol proxidase and catalase, and a slighter reduction in other antioxidant enzyme activity under cadmium treatment (Zawoznik et al. 2007). Salicylic acid seems to play a protective role in photosynthesis. Pea, maize, and wheat plants pretreated with SA and subjected to Cd stress showed a diminished reduction in chlorophyll content and/or photosynthetic enzymes activity (Krantev et al. 2008; Moussa and El-Gamal 2010a; Popova et al. 2009). However, in castor beans exposed to Cd, pretreatment with SA aggravated the toxic effect of this heavy metal on growth and photosynthetic parameters (Liu et al. 2011). These reports suggest that the mode of SA action depends on the concentration of salicylic acid and the plant’s susceptibility to this hormone.

Elevated levels of jasmonic acid has been observed in pea, runner bean, and *Arabidopsi*s plants treated with cadmium (Maksymiec et al. 2005; Rodríguez-Serrano et al. 2006). It is suggested that JA has a protective effect against Cd action at lower concentrations (Maksymie and Krupa 2002; Noriega et al. 2012). However, at higher concentrations, it may induce changes usually observed under heavy metal stress, such as growth reduction, chlorophyll degradation, and inhibition of various photosynthetic parameters (Maksymie and Krupa 2002). Jasmonic acid might also interact with ROS signaling—it has been shown to mediate the generation of reactive oxygen species in *Arabidopsis* plants treated with CdSO_4_ (Maksymiec and Krupa 2006).

The response of abscisic acid to cadmium is not so obvious. A decrease in ABA content has been observed in wheat plants treated with 400 and 1000 μM of Cd, while an increase in the hormone’s content has been reported in two rice cultivars subjected to 500 μM Cd. Interestingly, ABA induction was greater in Cd-tolerant rice cultivar (Hsu and Kao 2003; Moussa and El-Gamal 2010b). Exogenous addition of ABA resulted in enhanced tolerance to cadmium stress and a decrease in uptake of this heavy metal (Hsu and Kao 2003). Another piece of evidence for the protective role of abscisic acid against cadmium stress has been provided by a comparison of the reaction to CdSO_4_ of wild type *Arabidopsis* plants and ABA-deficient plants. The experiments showed that the mutants were more sensitive to Cd (Sharma and Kumar 2002). These facts suggest that ABA might be involved in signaling events that lead to a decrease in Cd accumulation or in the activation of other defense mechanisms against heavy metal stress. However, the exact role of ABA in cadmium signal transduction is still unknown.

## Conclusions

On the basis of the facts described above, it can be concluded that animal and plant cadmium signal transduction networks share many resemblances. Therefore, it is probable that at least some elements involved in cadmium sensing, are evolutionary conserved. These elements include mechanism of cadmium uptake, engagement of phospholipases and MAP kinases and involvement of ROS, Ca^2+^ and NO signaling molecules. It is also probable that both animals and plants posses in their genome metal responsive elements, that are engaged in regulation of expression of Cd-responsive genes. Except the common response, animals and plants posses also specific signaling pathways activated by cadmium. In case of animals Wnt/β-catenin, sonic hedgehog and oestrogen signaling can be enumerated. In plants, hormones such as ethylene and jasmonic, salicylic and abscisic acid are engaged in the transduction of cadmium signal. It can be therefore concluded, that cadmium influences most of the main animal and plant signaling pathways, the common as well as the specific ones.

## Abbreviations

ABA: Abscisic acid
AIF: Apoptosis-inducing factor
ACS: 1-aminocyclopropane-1-carboxylic acid synthase
AMPK: LKB1-AMP-activated kinase
AVG: Aminoethoxyvinylglycine
CAM: Calmodulin
CBL: Calcineurin B-like protein
CDPK: Calcium dependent protein-kinase
CLM: Calmodulin-like protein
DAG: Diacyloglycerol
ERK: Extracellular signal-regulated kinase
GSK-3β: Glycogen synthase kinase-3β
IP_3_: Inositol 1,4,5 triphosphate
JA: Jasmonic acid
JNK: *c-Jun* N-terminal kinase
LCT1: Low-affinity-cation transporter
LEF: Lymphoid enhancer binding factor
MAPK: Mitogen-activated protein kinase
MAPKK: Mitogen-activated protein kinase kinase
MAPKKK: Mitogen-activated protein kinase kinase kinase
MDCK: Madin-Darby canine kidney
MRE: Metal responsive element
MT: Methallothioneine
MTF: Metal-regulatory transcription factor, MRE-binding factor
NAC: N-acetylcysteine
NCS: Neuronal calcium sensor
NO: Nitric oxide
Nramp: Resistance-associated macrophage proteins
ROS: Reactive oxygen species
PAPR-1: Poly ADP-ribose polymerase-1
PI_3_: Phosphoinositol 3-kinase
PIP_3_: Phosphoinositol 4,5 biphosphate
PLC: Phospholipase C
PKC: Protein kinase C
SA: Salicylic acid
Shh: Sonic hedgehog
STS: Silver thiosulphate
TCF: T-cell specific factor
TF: Transcription factor
